# Serum type XVI collagen is associated with colorectal cancer and ulcerative colitis indicating a pathological role in gastrointestinal disorders

**DOI:** 10.1002/cam4.1692

**Published:** 2018-07-20

**Authors:** Christina Jensen, Signe H. Nielsen, Joachim H. Mortensen, Jens Kjeldsen, Lone G. Klinge, Aleksander Krag, Henrik Harling, Lars N. Jørgensen, Morten A. Karsdal, Nicholas Willumsen

**Affiliations:** ^1^ Biomarkers & Research Nordic Bioscience Herlev Denmark; ^2^ Biotech Research & Innovation Centre (BRIC) University of Copenhagen Copenhagen Denmark; ^3^ Department of Biotechnology and Biomedicine Technical University of Denmark Lyngby Denmark; ^4^ Department of Medical Gastroenterology Odense University Hospital Odense Denmark; ^5^ Digestive Disease Center Bispebjerg Hospital University of Copenhagen Copenhagen Denmark

**Keywords:** biomarkers, collagen, colorectal cancer, extracellular matrix, ulcerative colitis

## Abstract

Altered extracellular matrix (ECM) remodeling is an important part of the pathology of gastrointestinal (GI) disorders. In the intestine, type XVI collagen (col‐16) plays a role in pathogenesis by affecting ECM architecture and induce cell invasion. Measuring col‐16 in serum may therefore have biomarker potential in GI disorders such as colorectal cancer (CRC) and ulcerative colitis (UC). The aim of this study was to determine whether col‐16 can serve as a biomarker for altered ECM remodeling in patients with CRC and UC. A monoclonal antibody was raised against the C‐terminal end of col‐16 (PRO‐C16), and a competitive enzyme‐linked immunosorbent assay (ELISA) was developed and technically validated. Levels of PRO‐C16 were measured in serum from patients with CRC (before (n = 50) and 3 months after (n = 23) tumor resections), UC (n = 39) and healthy controls (n = 50). The PRO‐C16 ELISA was specific toward the C‐terminal of col‐16. PRO‐C16 was significantly elevated both in serum from patients with CRC (*P* = 0.0026) and UC (*P* < 0.0001) compared to controls. No difference was detected in levels of PRO‐C16 between patients with CRC at baseline and 3 months after tumor resections (*P* > 0.999). Levels of PRO‐C16 identified patients with a GI disorder with a positive predictive value of 0.9 and an odds ratio of 12 (95%CI = 4.5‐29.5, *P* < 0.0001). The newly developed assay detected significantly elevated levels of PRO‐C16 in serum from patients with GI disorders compared to controls suggesting its potential as a biomarker in this setting. Future studies are needed to validate these findings.

## 1 INTRODUCTION

The extracellular matrix (ECM) is a noncellular component responsible for maintaining tissue architecture. Altered ECM remodeling is a significant part of the pathology of gastrointestinal (GI) disorders such as colorectal cancer (CRC)^1^ and ulcerative colitis (UC).^2^ An imbalance between ECM formation and degradation in the colon leads to an altered composition of the ECM causing abnormal tissue function. Elevated deposition of ECM proteins in the tumor microenvironment increases the stiffness of the ECM, which influences cellular functions such as cell proliferation, adhesion, migration, and invasion.^3^, ^4^ It has also become evident that inflammatory responses in the tumor microenvironment affect ECM remodeling.^5^, ^6^ Likewise, in UC, the ECM of the intestine is highly affected by chronic inflammation which leads to loss of tissue homeostasis and imbalanced collagen turnover.^2^, ^7^, ^8^, ^9^ The chronic inflammation and the continuous turnover of epithelial cells contribute to development of dysplasia which may transform into CRC.^10^ Biomarkers reflecting this enhanced ECM remodeling may therefore be important to identify patients with disruption in tissue/ECM architecture responsible for development and progression of CRC and UC.

In the intestine, type XVI collagen (col‐16) is suggested to contribute to stabilization and maintenance of basement membranes, a specialized layer of ECM located beneath the epithelial and endothelial cell layers.^11^ Col‐16 is a fibril‐associated collagen with interrupted triple helices (FACITs), and expressed by epithelial cells and subepithelial myofibroblasts. These are localized subjacent to the basement membrane with a pronounced deposition of col‐16 into the matrix of the epithelial crypts.^11^ Studies of skin show that col‐16 is localized in the dermal‐epidermal junction zone near basement membranes, which suggests that col‐16 has an active role in anchoring microfibrils to basement membranes.^12^, ^13^

**Figure 1:**
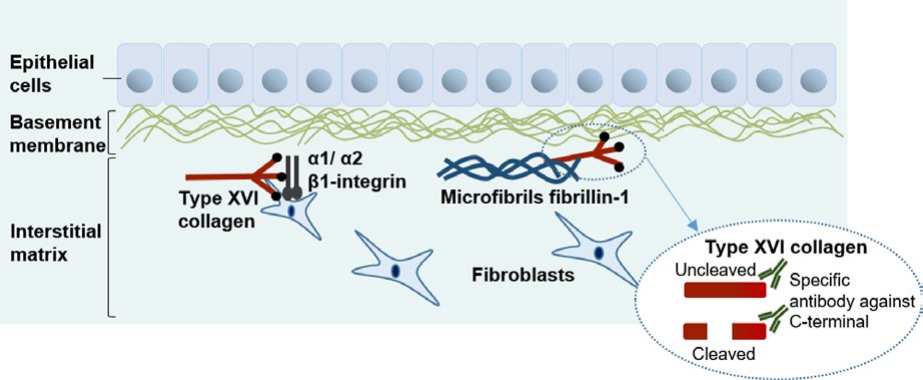
Type XVI collagen in the extracellular matrix. Type XVI collagen (col‐16) is a physiological binding partner of integrins α1/α2β1 where it induces integrin‐mediated cell reactions such as cell spreading. Col‐16 binds to macromolecules of the extracellular matrix (ECM) as fibrillin‐1 positive microfibrils. In this study, we target the C‐terminal of col‐16 with a specific antibody

Col‐16 interacts with α1β1 and α2β1 integrins and induces the recruitment of these integrins into focal adhesion plaques, which promote integrin‐mediated cell reactions, such as cell spreading and alterations in cell morphology (Figure 1).^14^, ^15^ The binding of col‐16 to integrins stimulates cell‐matrix interactions, which may induce an invasive phenotype in tumor cells. Interestingly, overexpression of col‐16 has been shown to induce cell invasion and a proliferative cellular phenotype in oral squamous cell cancer (OSCC).^16^, ^17^ Col‐16 is deposited at the basement membrane in normal oral epithelium while it seems to disappear from the basement membrane in tissues from OSCC patients.^17^ The loss of col‐16 from the basement membrane zone in the development of OSCC may induce ECM remodeling and a disruption of the basement membrane that promotes tumor cell infiltration and a progression of disease. In glioblastomas, col‐16 is involved in tumor cell adhesion and invasion as well as tumor‐specific remodeling of the ECM.^18^, ^19^ Increased expression of col‐16 has also been detected in intestinal subepithelial myofibroblasts isolated from inflamed Crohn's disease tissue biopsies.^11^

Despite the observation of enhanced expression of col‐16 concurrent to increasing inflammation in the intestine, the expression levels of col‐16 in UC and CRC patients remain unclear.

We hypothesized that col‐16 fragments may be released into the circulation as a part of GI pathogenesis and may therefore have potential as a biomarker in CRC and UC reflecting enhanced ECM remodeling associated with inflammation. Therefore, we developed a competitive enzyme‐linked immunosorbent assay (ELISA) targeting a sequence identical to the C‐terminal of col‐16, followed by evaluation of its levels in serum from patients with CRC and UC.

## 2 MATERIALS AND METHODS

### 2.1 Reagents

All reagents used for the experiments were standard chemicals from Merck (Whitehouse station, NJ, USA) and Sigma‐Aldrich (St. Louis, MO, USA). The synthetic peptides used for antibody production and assay development were purchased from the Chinese Peptide Company (Beijing, China) (Table 1).

**Table 1:** Synthetic peptides used for antibody production and assay development

Peptide	Sequence
Selection peptide	PMKTMKGPFG
Immunogenic peptide	KLH‐CGG‐PMKTMKGPFG
Biotinylated peptide	Biotin‐K‐PMKTMKGPFG
Elongated peptide	PMKTMKGPFGG
Nonsense peptide	VPKDLPPDTT
Nonsense biotinylated peptide	Biotin‐VPKDLPPDTT

KLH, Keyhole Limpet Hemocyanin.

### 2.2 Selection and overview of peptides

We chose to target the C‐terminal of the α1 chain of col‐16 and name this PRO‐C16. The amino acid sequence 1595′‐PMKTMKGPFG‐′1604 located at the C‐terminal was used to generate an antibody specific for the C‐terminal of col‐16. It was additionally used to design the selection peptide (PMKTMKGPFG) (Table 1). The sequence was blasted for homology to other human proteins and species using the NPS@: network protein sequence analysis with the Uniprot/Swiss‐Prot database.^20^ The amino acid sequence is unique to human col‐16. A biotinylated peptide (Biotin‐K‐PMKTMKGPFG) was used to coat the streptavidin‐coated plates applied in the ELISA. An elongated peptide (PMKTMKGPFGG), a nonsense peptide (VPKDLPPDTT), and a nonsense biotinylated peptide (Biotin‐VPKDLPPDTT) were included to test the specificity of the antibody.

### 2.3 Monoclonal antibody production and clone characterization

Generation of monoclonal antibodies was carried out as previously described.^21^ Briefly, four‐ to six‐week‐old Balb/C mice were immunized subcutaneously with 200 μL emulsified antigen and 50 μg immunogenic peptide (Keyhole Limpet Hemocyanin (KLH)‐CGG‐PMKTMKGPFG) using Freund's incomplete adjuvant (Sigma‐Aldrich). The mice were immunized with two‐week intervals until stable serum titer levels were reached. The mouse with the highest serum titer was selected for fusion. The mouse rested one month was immunized intravenously with 50 μg immunogenic peptide in 100 μL 0.9% sodium chloride (NaCl) solution. After 3 days, splenocytes were isolated for cell fusion. In brief, splenocytes were fused with SP2/0 myeloma cells to produce hybridoma cells and then cloned in culture dishes using the semi‐medium method.^22^ The clones were plated into 96‐well microtiter plates, and limited dilution was used to secure monoclonal growth. The supernatants were screened for reactivity against the selection peptide (PMKTMKGPFG) and native material (serum) in an indirect competitive ELISA using streptavidin‐coated plates (Roche, Hvidovre, Denmark, cat. 11940279). The clones with the best reactivity were purified using protein‐G‐columns according to the manufacturer's instructions (GE healthcare Life Sciences, Little Chalfont, Buckinghamshire, UK). Two monoclones were tested for their reactivity toward the selection peptide (PMKTMKGPFG) and not the elongated (PMKTMKGPFGG) or nonsense peptide (VPKDLPPDTT). One monoclone was chosen for assay development. Optimal incubation buffer, time, temperature, and optimal ratio between the biotinylated peptide and antibody were determined.

### 2.4 PRO‐C16 ELISA protocol

The competitive ELISA procedure was as follows: a 96‐well streptavidin‐coated microtiter plate was coated with 100 μL of biotinylated peptide (Biotin‐K‐PMKTMKGPFG) dissolved in assay buffer (50 mmol/L phosphate‐buffered saline with bovine serum albumin (1% w/v), Tween‐20 (0.1% w/v), and bronidox (0.36% v/v) (PBS‐BTB), 4 g/L NaCl, pH 7.4) (final concentration of 3.1 ng/mL). The plate was incubated for 30 minutes at 20°C with shaking (300 rpm) and then washed five times in washing buffer (20 mmol/L TRIS, 50 mmol/L NaCl, pH 7.2). A volume of 20 μL of sample/control/selection peptide (PMKTMKGPFG) was added followed by immediately addition of 100 μL of monoclonal antibody diluted in assay buffer (final concentration of 62.5 ng/mL). The plate was incubated for 1 hour at 20°C with shaking followed by five washes in washing buffer. Then, 100 μL of goat anti‐mouse horseradish peroxidase (HRP)‐conjugated IgG antibody (Thermo Scientific, Waltham, MA, USA; cat. #31437) diluted in assay buffer (final concentration of 130 ng/mL) was added to each well. The plate was incubated for 1 hour at 20°C with shaking and subsequently washed five times in washing buffer. Next, 100 μL Tetramethylbenzidine (TMB, Kem‐En‐Tec Diagnostics, Taastrup, Denmark) was added and incubated for 15 minutes at 20°C with shaking in the dark. To stop the reaction of TMB, 100 μL of 1% sulfuric acid (H_2_SO_4_) was added and the plate was analyzed in a VersaMax ELISA microplate reader at 450 nm with 650 nm as reference. A standard curve was plotted using a 4‐parametric mathematical fit model, and data were analyzed using the Softmax Pro v. 6.3 software.

### 2.5 Technical evaluation

Antibody specificity was calculated as percentage of signal inhibition of twofold diluted selection peptide (PMKTMKGPFG), elongated peptide (PMKTMKGPFGG), or nonsense peptide (VPKDLPPDTT). Lower limit of measurement range (LLMR) and upper limit of measurement range (ULMR) were calculated based on the standard curve from 10 independent runs. A twofold dilution of healthy serum samples from humans (n = 3) was used to determine linearity and calculated as percentage recovery of the undiluted sample. Ten independent runs of seven samples that covered the detection range (LLMR‐ULMR) of the PRO‐C16 were used to calculate the intra‐ and‐interassay variation. The seven samples included three human serum samples and four samples with selection peptide spiked in assay buffer. The intra‐assay variation was determined as the mean coefficient of variance (CV%) within plates, and the interassay variation was calculated as the mean CV% between plates. Accuracy was determined from three human serum samples spiked with twofold dilutions of the selection peptide and calculated as percentage recovery of the expected concentration (serum and peptide combined). The analyte stability was determined for three healthy serum samples subjected to up to four freeze and thaw cycles. The freeze‐thaw recovery was calculated with the first cycle as reference. Analyte stability was furthermore determined by incubation of three human serum samples at either 4°C or 20°C for 24 or 48 hours. Recovery was calculated with samples stored at −20°C as reference. Interference was determined by adding low/high content of biotin (1.5/4.5 ng/mL), lipid (0.75/2.5 mg/mL) or hemoglobin (1.25/2.5 mg/mL) to serum samples of known concentrations and calculated as the percentage recovery of analyte in nonspiked serum.

### 2.6 Patient serum samples

Serum samples from CRC patients were collected by medical staff at Bispebjerg Hospital, Copenhagen, Denmark subsequent to informed consent and approval by the Ethical Committee of the Capital Region of Denmark (Copenhagen, Denmark; approval no. H‐1‐2014‐048) in compliance with the Helsinki Declaration. Both men and females were included in the study. Patients were excluded if: (a) they were under 18 years of age, (b) they were pregnant, (c) they were diagnosis with a psychotic disorder or dementia, (d) they had received prior chemotherapy or other cancer therapy. Serum samples were collected before (baseline) and 3 months after tumor resections (month 3) from 50 and 23 patients, respectively. The main reason for the reduced patient number 3 months after tumor resection was that patients did not show up to this voluntary control visit. Tumor staging was evaluated according to the Union for International Cancer Control classification system.

Serum samples from UC patients (n = 39) were obtained from Odense University Hospital (Odense, Denmark) after informed consent. The study was approved by the local ethics committee of Southern Denmark (S‐20070072) and the Danish Data Protection Agency (2007‐41‐0675). Patients were included if: (a) they had known UC, (b) they had at least one previous flare of clinical and endoscopic active disease, (c) age 18 and above. Exclusion criteria were as follows: (a) changes in azathioprine dosage within the past 3 months, (b) patients with toxic megacolon, (c) peritonitis or severe colonic bleeding, (d) known immunodeficiency, (e) ongoing infectious disease, (f) ongoing treatment with nonsteroidal anti‐inflammatory drugs or cholestyramine, (g) pregnant or lactating women.^9^ Levels of PRO‐C16 in the CRC and UC patients were compared to levels in commercially available control sera from healthy donors (n = 50) (Valley BioMedical, Winchester, VA, USA) who according to manufacturer's information all filed informed consent. Information associated with the included patients is shown in Table 2.

**Table 2:** Clinical characteristics of the study population

Clinical parameter	Controls n = 50	Colorectal cancer Baseline n = 50	Colorectal cancer Month 3 n = 23	Ulcerative colitis n = 39
Median age years (range)	51 (19‐85)	71 (32‐90)	70 (32‐83)	32 (22‐62)
Gender (% females)	8%	48%	43.5%	58.3%
Tumor stage				
I	—	7	5	—
II	—	27	13	—
III	—	9	3	—
IV	—	4	2	—
N/A	—	3	—	—
Treatment				
No adjuvant treatment	—	24	14	—
Adjuvant treatment (chemotherapy)	—	17	8	—
N/A	—	9	1	—
St. Mark score, median (range)	—	—	—	3 (0‐6)

### 2.7 Statistical analyses

Kruskal‐Wallis test was used to compare serum levels of PRO‐C16 in controls, CRC patients at baseline and UC patients. Wilcoxon test was used to compare CRC patients at baseline and 3 months after tumor resection. The odds ratio, sensitivity, specificity, and negative and positive predictive values were generated from a specific cutoff value, obtained from the area under the receiver operating characteristics (AUROC) curve, and analyzed using Fisher's exact probability test and chi‐square test. A *P*‐value of *P* < 0.05 was considered statistical significant. GraphPad Prism, version 7.01 (GraphPad Software, San Diego, CA, USA) was used for all statistical analyses.

## 3 RESULTS

### 3.1 Specificity of the PRO‐C16 assay

**Figure 2:**
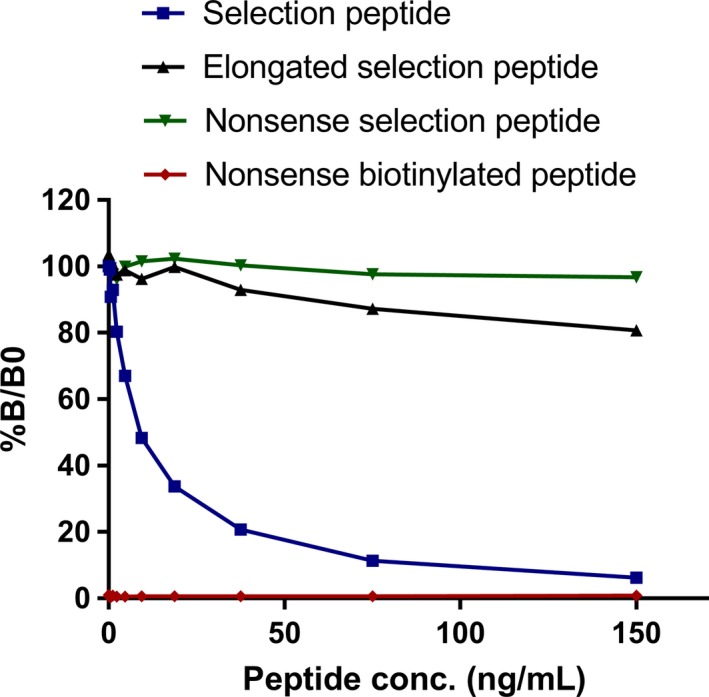
Specificity of the PRO‐C16 assay. The percentage of inhibition at given concentrations in the competitive PRO‐C16 ELISA tested with the selection peptide (PMKTMKGPFG), an elongated peptide (PMKTMKGPFGG), a nonsense peptide (VPKDLPPDTT), and a nonsense biotinylated peptide (Biotin‐K‐PMKTMKGPFG). %*B*/*B*0: *B* equals the OD at × nmol/L peptide and *B*0 equals the OD at 0 nmol/L peptide

The specificity of the newly developed PRO‐C16 ELISA was evaluated by investigating the inhibitory effect of different peptides. The selection peptide (PMKTMKGPFG) inhibited the signal to 6% at the highest concentration while an elongated peptide (PMKTMKGPFGG) and a nonsense peptide (VPKDLPPDTT) inhibited the signal to 81% and 97%, respectively (Figure 2). No reactivity was observed toward a nonsense biotinylated peptide (Biotin‐VPKDLPPDTT). Altogether, this indicates that the antibody is specific to a peptide sequence identical to the C‐terminal of col‐16.

### 3.2 Technical evaluation of the PRO‐C16 assay

Several tests were included to evaluate the overall technical performance of the PRO‐C16 assay (Table 3). The measurement range was determined by calculating the LLMR and ULMR, which provided a range of 0.87‐95.50 ng/mL. Intra‐ and interassay variation was 10% and 15%, respectively. Native reactivity was observed in human serum. The dilution recovery in serum was 95% observed from undiluted to a 1:4 dilution. Spiking of standard peptide in human serum resulted in a mean recovery of 99%, indicating accuracy and that sample matrix do not affect assay response. The stability of the analyte was acceptable after four freeze‐thaw cycles with a 103% recovery. The analyte was also recovered after prolonged storage of human serum at 4°C for 24 or 48 hours, resulting in a 106% and 95% recovery, respectively. Storage at 20°C for 24 or 48 hours resulted in a 91% and 85% recovery, respectively. No interference was detected from either low or high levels of biotin, lipids, or hemoglobin.

**Table 3:** Technical validation of the PRO‐C16 assay

Technical validation step	Results
Detection range (LLMR‐ULMR)	0.87‐95.50 ng/mL
Intra‐assay variation	10%
Inter‐assay variation	15%
Dilution recovery in serum	95%
Spiking recovery in serum	99%
Freeze‐thaw recovery in serum	103%
Analyte stability in serum 24 h, 4°C/20°C	106%/91%
Analyte stability in serum 48 h, 4°C/20°C	95%/85%
Interference	
Recovery in Biotin low/high	94%/113%
Recovery in Lipid low/high	137%/118%
Recovery in Hemoglobin low/high	97%/100%

LLMR, lower limit of measurement range; ULMR, upper limit of measurement range. Percentages are reported as mean.

### 3.3 Serum PRO‐C16 levels are higher both in patients with colorectal cancer and ulcerative colitis compared to healthy controls

**Figure 3:**
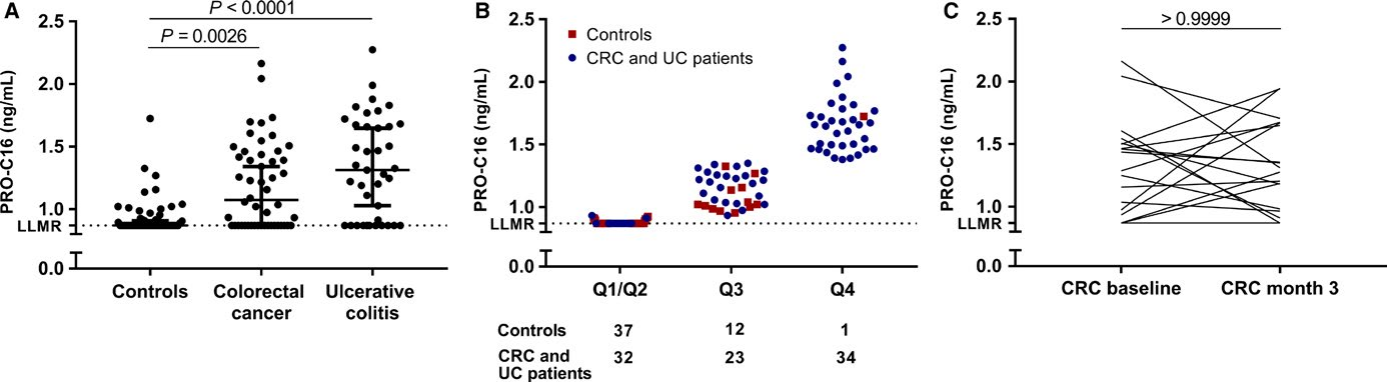
Serum PRO‐C16 levels are higher both in patients with colorectal cancer (CRC) and ulcerative colitis (UC) compared to healthy controls. A, PRO‐C16 levels in serum from controls (n = 50), CRC (n = 50), and UC patients (n = 39). Levels below lower limit of measurement range (LLMR) are adjusted to LLMR. Error bars represent the median ± 95%CI of the patients measured in duplicates. Groups were compared using Kruskal‐Wallis test. B, Levels of PRO‐C16 in serum from CRC patients, UC patients, and controls divided by quartiles (Q). Patients with levels below the median (Q1/Q2), range 0.87‐0.93 ng/mL. Patients with levels above the median and under the upper quartile (Q3), range 0.93‐1.35 ng/mL. Patients with levels in the upper quartile (Q4), range 1.38‐2.27 ng/mL. The number of controls, CRC, and UC patients in each group is illustrated. C, PRO‐C16 levels were compared in serum from CRC patients at baseline and 3 months after tumor resections (month 3). Statistically significant difference was determined using the paired Wilcoxon test

**Figure 4:**
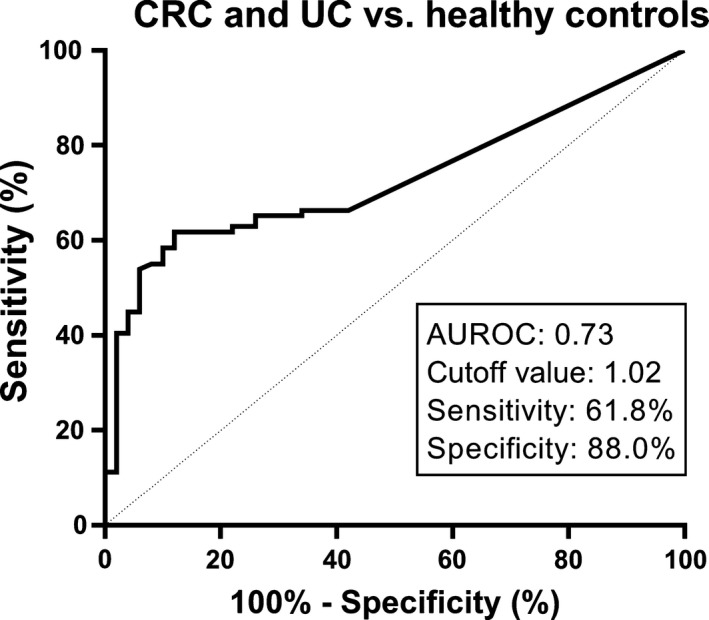
Receiver operating characteristics (ROC) analysis. ROC curve analysis was used to evaluate the ability of PRO‐C16 to discriminate between CRC and UC patients and healthy controls

To determine the biomarker potential of col‐16, we measured PRO‐C16 levels both in serum from patients with CRC and UC compared to healthy controls. PRO‐C16 levels were significantly elevated in patients with CRC (1.07 ng/mL, 95%CI = 0.87‐1.34, *P* = 0.0026) and UC (1.31 ng/mL, 95%CI = 1.03‐1.65, *P* < 0.0001) compared to healthy controls (0.87 ng/mL, 95%CI = 0.87‐0.91) (Figure 3A). The percentage of CRC and UC cases of the total tested population increased stepwise with increasing quartile (Figure 3B). Of the population with PRO‐C16 levels in the upper quartile (Q4), 97% (34/35) were CRC or UC patients while 3% (1/35) were healthy controls. PRO‐C16 was able to identify patients with a GI disorder (CRC + UC) with a positive predictive value of 0.9 and an odds ratio of 12 (95%CI = 4.5‐29.5, *P* < 0.0001). The negative predictive value was 0.6. The diagnostic power (AUROC) of PRO‐C16 for a patient suffering from a GI disorder compared to healthy controls was 0.73 (95%CI = 0.64‐0.81, *P* < 0.0001). The ROC curve, as well as the sensitivity and specificity are shown in Figure 4. Thus, measuring PRO‐C16 in serum has biomarker potential in GI disorders.

When PRO‐C16 levels were compared (paired) between the CRC patients before tumor resections (baseline) and 3 months after tumor resections (month 3), no difference was observed (*P* > 0.9999) (Figure 3C). In addition, when dividing the patients into two groups: those receiving adjuvant treatment and those not receiving treatment, still no difference could be detected in PRO‐C16 levels at the two timepoints, suggesting that the PRO‐C16 levels are not affected by this treatment (data not shown). This indicates that col‐16 does not originate from the primary tumor.

**Figure 5:**
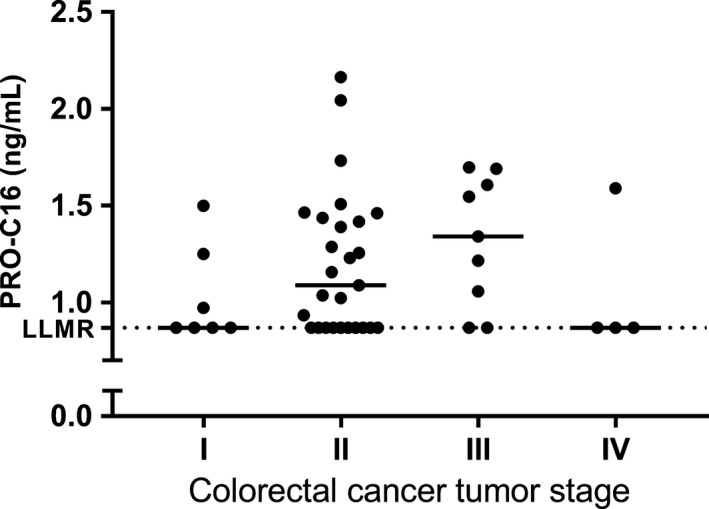
Evaluation of PRO‐C16 in serum from colorectal cancer (CRC) patients separated by tumor stage. Levels of PRO‐C16 in serum from CRC patients at baseline divided into stage of disease with the median illustrated by a horizontal line. Groups were compared using Kruskal‐Wallis test

As the tumor stage is an important clinical tool in CRC, the PRO‐C16 levels were divided according to tumor stage (Figure 5). No significant difference was detected between the tumor stages. However, a trend was observed for elevated levels of PRO‐C16 in stages II and III.

## 4 DISCUSSION

In the present study, we developed and validated a robust competitive ELISA that enables noninvasive measurement of PRO‐C16. Using our PRO‐C16 assay, we observed significantly elevated levels of PRO‐C16 both in serum from patients with CRC and UC compared to healthy controls. Of note, no difference in PRO‐C16 levels was observed after tumor resections compared to baseline. To our knowledge, this is the first study to show that PRO‐C16 can be measured in serum and that PRO‐C16 has biomarker potential for GI disorders.

The observation that PRO‐C16 was elevated in both UC and CRC suggests a link between col‐16 and diseases of the intestine, which has been described previously.^11^ In support of this, the similar PRO‐C16 levels in serum from CRC patients before and 3 months after tumor resections propose that col‐16 does not only originate from the primary tumor but also other places in the colon where it may rather reflect an unhealthy GI phenotype; PRO‐C16 levels may be indicative of pathological events in the colon.

Increased levels of col‐16 have been demonstrated in inflamed intestinal tissue.^11^ It could be speculated that the elevated expression of col‐16 has a role during progression and/or maintenance of inflammation in the inflamed tissue.^11^ Data implicates that the increased col‐16 in inflamed colon tissue induces a prolonged adhesion of intestinal subepithelial myofibroblasts to the underlying matrix.^11^ Increased col‐16 would therefore help to promote a pathological maintenance of the myofibroblasts at the inflammation site causing continuous fibrotic responses with excessive accumulation of ECM components.^23^ Thus, col‐16 could be implicated in the pathogenesis of fibrostenotic phenotypes in inflammatory bowel diseases.

Upregulation of col‐16 has previously been identified as a potential mediator of localized mechanical stiffness in high mammographic density tissue.^24^ Altered ECM remodeling is an important part of cancer development, and excessive collagen turnover products are released during CRC progression.^1^, ^25^ Consistent with this, we observed increased levels of PRO‐C16 in serum from patients with CRC (Figure 3A). In the tumor microenvironment, increased col‐16 may induce prolonged adhesion of fibroblasts, which leads to deposition of collagen and tissue stiffness associated with CRC progression.^26^ Col‐16 is upregulated by transforming growth factor‐β (TGF‐β), and high levels of TGF‐β have been shown to be associated with metastasis and poor outcome in CRC patients.^27^, ^28^ As the tumor stroma plays a crucial role in tumor progression, there are increasing efforts in developing anti‐fibrotic drugs targeting the stroma in cancer patients.^29^ It could be speculated that anti‐fibrotic treatments also are going to be tested in CRC patients and biomarkers reflecting changes in the ECM would therefore be essential.

A trend was observed for elevated levels of PRO‐C16 in patients with stages II and III CRC compared to stage I suggesting that col‐16 may have a role in cell invasion. Col‐16 has shown to promote formation of focal adhesion contacts and cell spreading in colon tissue.^11^ In addition, col‐16 has previously shown to induce cell invasion in OSCC and glioblastomas.^16^, ^18^, ^19^ Col‐16 induces matrix metalloproteinase 9 (MMP9) expression, which modulates the tumor microenvironment and has been associated with the acquisition of an invasive phenotype in many tumors.^16^ Therefore, we would also expect higher PRO‐C16 levels in stage IV. The low level of PRO‐C16 at stage IV is calculated from four patients. Thus, further studies are needed to validate these observations.

The monoclonal antibody was shown to be specific toward an amino acid sequence identical to the C‐terminal of col‐16. The C‐terminal contains the NC1 domain, which of other collagens has shown to exhibit signaling properties and have biomarker potential.^30^, ^31^ Therefore, we found it relevant to raise this novel monoclonal antibody for use in an ELISA instead of using commercially available polyclonal antibodies. One limitation is that the assays’ antibody specificity only could be investigated for the target peptide sequence used to generate the monoclonal antibody, or other recombinant derivatives of col‐16 with similar limitations, that is, a non‐native col‐16 fragment.

Technical evaluation of PRO‐C16 included the establishment of detection range, inter‐ and intra‐assay variation, dilution recovery, analyte stability, and interference, which all were within prespecified acceptable limits (Table 3). Moreover, when spiking the analyte into serum and calculating the percentage recovery, this was also within acceptable limits indicating that the assay has accuracy toward the analyte of interest in serum (Table 3). In summary, the PRO‐C16 assay was technically robust, specific, and accurate.

The present study is limited by the available clinical information and the fact that age and gender could be confounding factors. Hence, it will be desirable to study the level of PRO‐C16 in age‐ and gender‐matched samples and with more clinical information available.

In conclusion, PRO‐C16 can be quantified in serum by the technically robust and specific PRO‐C16 assay. Our study demonstrates significantly elevated PRO‐C16 levels in CRC and UC patients compared to healthy controls, suggesting a pathological role of col‐16 in the colon. Future studies will determine the exact role and biomarker potential of PRO‐C16 in GI disorders.
